# *catena*-Poly[[(2,2′:6′,2′′-terpyridine-κ^3^ *N*,*N*′,*N*′′)zinc(II)]-μ-2,2′-oxydibenzo­ato-κ^2^ *O*:*O*′]

**DOI:** 10.1107/S1600536809047679

**Published:** 2009-11-14

**Authors:** Hai-Yan Gong, Yan Bai, Wei Liu

**Affiliations:** a The Center of Analysis and Determination, Henan University of Traditional Chinese Medicine, No. 1 Jinshui Road, Zhengzhou 450008, Henan, People’s Republic of China

## Abstract

In the title compound, [Zn(C_14_H_8_O_5_)(C_15_H_11_N_3_)]_*n*_, both the Zn^II^ ion and the oxydibenzoate ligand are located on a twofold rotation axis. The Zn^II^ centre is coordinated by three N atoms from a chelating 2,2′:6′,2′′-terpyridine ligand and two O atoms from two 2,2′-oxydibenzoate ligands, forming a distorted trigonal-bipyramidal coordination environment. Further coordination *via* the 2,2′-oxydibenzoate anions forms a one-dimensional coordination polymer extending parallel to [010]. Aromatic π–π stacking inter­actions are observed between adjacent terpyridine ligands with a centroid–centroid distance of 3.568 (2) Å.

## Related literature

For related structures, see: Zhao & Li (2009 ▶); Andres & Schubert (2004 ▶); Constable (1986 ▶); Hofmeier & Schubert (2004 ▶).
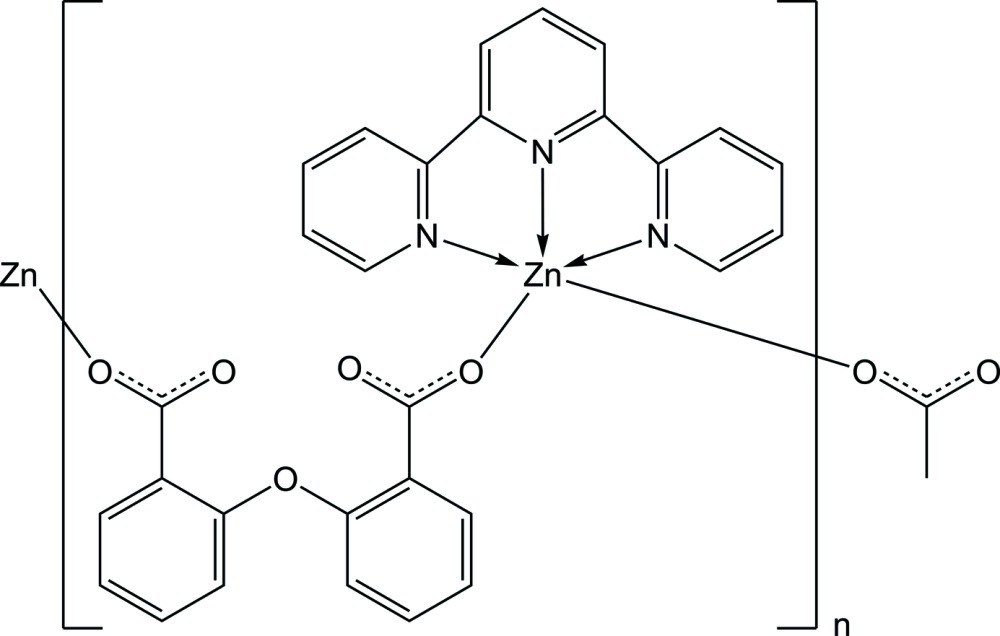


## Experimental

### 

#### Crystal data

[Zn(C_14_H_8_O_5_)(C_15_H_11_N_3_)]
*M* *_r_* = 554.84
Orthorhombic, 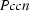

*a* = 8.7985 (17) Å
*b* = 10.694 (2) Å
*c* = 25.535 (5) Å
*V* = 2402.6 (8) Å^3^
*Z* = 4
Mo *K*α radiation
μ = 1.07 mm^−1^
*T* = 296 K
0.20 × 0.18 × 0.16 mm

#### Data collection

Bruker SMART APEXII CCD area-detector diffractometer
Absorption correction: multi-scan (*SADABS*; Bruker, 2007 ▶) *T*_min_ = 0.815, *T*_max_ = 0.847
15388 measured reflections
2924 independent reflections
2347 reflections with *I* > 2σ(*I*)
*R*_int_ = 0.043

#### Refinement

*R*[*F* ^2^ > 2σ(*F* ^2^)] = 0.046
*wR*(*F* ^2^) = 0.098
*S* = 1.08
2924 reflections
174 parameters
H-atom parameters constrained
Δρ_max_ = 0.39 e Å^−3^
Δρ_min_ = −0.31 e Å^−3^

### 

Data collection: *APEX2* (Bruker, 2007 ▶); cell refinement: *SAINT* (Bruker, 2007 ▶); data reduction: *SAINT*; program(s) used to solve structure: *SHELXTL* (Sheldrick, 2008 ▶); program(s) used to refine structure: *SHELXTL*; molecular graphics: *DIAMOND* (Brandenburg, 1999 ▶); software used to prepare material for publication: *SHELXTL*.

## Supplementary Material

Crystal structure: contains datablocks I, global. DOI: 10.1107/S1600536809047679/nk2011sup1.cif

Structure factors: contains datablocks I. DOI: 10.1107/S1600536809047679/nk2011Isup2.hkl

Additional supplementary materials:  crystallographic information; 3D view; checkCIF report

## Figures and Tables

**Fig. 1.**
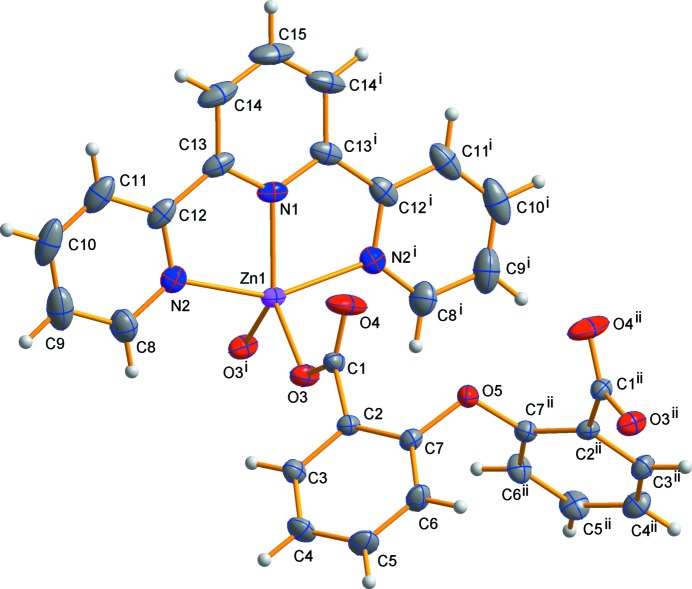
The molecular structure of the title compound with the atom-labelling scheme. Displacement ellipsoids are drawn at the 30% probability level. H atoms are presented as small spheres of arbitrary radius. [Symmetry codes: (i) -x+1/2, -y+1/2, z; (ii) -x+1/2, -y-1/2, z.]

## supplementary crystallographic information

### Comment

2,2':6',2''-Terpyridine and its derivatives have been intensively explored because of the interesting electronic, photonic, magnetic, reactive and structural properties shown by the transition metal complexes of these ligands (Andres & Schubert, 2004; Constable, 1986; Hofmeier & Schubert, 2004). We report here the synthesis and structure of the Zn^II^ complex based on the 2,2':6',2''-terpyridine ligand.

In the crystal structure of the title compound, the Zn atoms are coordinated by three N atoms from a chelating terpy ligand and two O atoms from two 2,2'-oxydibenzoate ligands, forming a distorted trigonal bipyramidal coordination environment (Figure 1). The Zinc atoms are linked by the 2,2-oxydibenzoate anions into a one-dimensional coordination polymer.

Aromatic stacking interactions between *Cg*1 and *Cg*2 [*Cg*1 and *Cg*2 are (N2, C8 – C12) and (N2^i^, C8^i^ – C12^i^) ring centroids, respectively, symmetry code:(i) -1/2 - *x*,1/2 - *y*,*z*] are observed, with a centroid–centroid distances of 3.568 (2) Å.

### Experimental

The title complound was synthesized hydrothermally in a Teflon-lined autoclave (25 ml) by heating a mixture of 2,2':6',2''-terpyridine (0.2 mmol), 2,2'-oxydibenzoic acid (0.4 mmol) and ZnSO_4_.H_2_O (0.2 mmol) in water (10 ml) at 393 K for 3 d. The autoclave was slowly cooled to room temperature. Crystals suitable for X-ray analysis were obtained.

### Refinement

All the H atoms could be detected in the difference Fourier map. Nevertheless, they were situated into the idealized position and refined using a C—H = 0.93 Å and *U*_iso_(H) = 1.2*U*_eq_(C).

### Crystal data

**Table:**

[Zn(C_14_H_8_O_5_)(C_15_H_11_N_3_)]	*F*(000) = 1136
*M**_r_* = 554.84	*D*_x_ = 1.534 Mg m^−^^3^
Orthorhombic, *P**c**c**n*	Mo *K*α radiation, λ = 0.71073 Å
Hall symbol: -P 2ab 2ac	Cell parameters from 2235 reflections
*a* = 8.7985 (17) Å	θ = 2.1–25.4°
*b* = 10.694 (2) Å	µ = 1.07 mm^−^^1^
*c* = 25.535 (5) Å	*T* = 296 K
*V* = 2402.6 (8) Å^3^	Block, colourless
*Z* = 4	0.20 × 0.18 × 0.16 mm

### Data collection

**Table:**

Bruker SMART APEXII CCD area-detector diffractometer	2924 independent reflections
Radiation source: sealed tube	2347 reflections with *I* > 2σ(*I*)
graphite	*R*_int_ = 0.043
ω scans	θ_max_ = 28.1°, θ_min_ = 1.6°
Absorption correction: multi-scan (*SADABS*; Bruker, 2007)	*h* = −11→11
*T*_min_ = 0.815, *T*_max_ = 0.847	*k* = −10→14
15388 measured reflections	*l* = −27→33

### Refinement

**Table:**

Refinement on *F*^2^	Primary atom site location: structure-invariant direct methods
Least-squares matrix: full	Secondary atom site location: difference Fourier map
*R*[*F*^2^ > 2σ(*F*^2^)] = 0.046	Hydrogen site location: inferred from neighbouring sites
*wR*(*F*^2^) = 0.098	H-atom parameters constrained
*S* = 1.08	*w* = 1/[σ^2^(*F*_o_^2^) + (0.0319*P*)^2^ + 2.226*P*] where *P* = (*F*_o_^2^ + 2*F*_c_^2^)/3
2924 reflections	(Δ/σ)_max_ < 0.001
174 parameters	Δρ_max_ = 0.39 e Å^−^^3^
0 restraints	Δρ_min_ = −0.31 e Å^−^^3^

### Special details

**Table:**

Geometry. All e.s.d.'s (except the e.s.d. in the dihedral angle between two l.s. planes) are estimated using the full covariance matrix. The cell e.s.d.'s are taken into account individually in the estimation of e.s.d.'s in distances, angles and torsion angles; correlations between e.s.d.'s in cell parameters are only used when they are defined by crystal symmetry. An approximate (isotropic) treatment of cell e.s.d.'s is used for estimating e.s.d.'s involving l.s. planes.
Refinement. Refinement of *F*^2^ against ALL reflections. The weighted *R*-factor *wR* and goodness of fit *S* are based on *F*^2^, conventional *R*-factors *R* are based on *F*, with *F* set to zero for negative *F*^2^. The threshold expression of *F*^2^ > σ(*F*^2^) is used only for calculating *R*-factors(gt) *etc*. and is not relevant to the choice of reflections for refinement. *R*-factors based on *F*^2^ are statistically about twice as large as those based on *F*, and *R*- factors based on ALL data will be even larger.

### Fractional atomic coordinates and isotropic or equivalent isotropic displacement parameters (Å^2^)

**Table:**

	*x*	*y*	*z*	*U*_iso_*/*U*_eq_	
C1	0.1521 (3)	0.0127 (2)	0.37317 (9)	0.0328 (5)	
C2	0.0873 (3)	−0.0769 (2)	0.33333 (8)	0.0279 (5)	
C3	−0.0284 (3)	−0.0330 (2)	0.30090 (10)	0.0371 (6)	
H3	−0.0602	0.0495	0.3044	0.045*	
C4	−0.0974 (3)	−0.1075 (3)	0.26391 (11)	0.0461 (7)	
H4	−0.1743	−0.0756	0.2428	0.055*	
C5	−0.0514 (3)	−0.2295 (3)	0.25857 (11)	0.0484 (7)	
H5	−0.0971	−0.2807	0.2337	0.058*	
C6	0.0626 (3)	−0.2760 (2)	0.29012 (11)	0.0428 (6)	
H6	0.0930	−0.3589	0.2866	0.051*	
C7	0.1323 (3)	−0.2002 (2)	0.32707 (9)	0.0311 (5)	
C8	−0.0782 (4)	0.3622 (3)	0.39283 (15)	0.0611 (9)	
H8	−0.0598	0.3578	0.3570	0.073*	
C9	−0.2154 (4)	0.4118 (4)	0.4104 (2)	0.0781 (12)	
H9	−0.2872	0.4412	0.3866	0.094*	
C10	−0.2425 (4)	0.4166 (3)	0.4628 (2)	0.0807 (13)	
H10	−0.3337	0.4489	0.4752	0.097*	
C11	−0.1361 (4)	0.3741 (3)	0.49663 (16)	0.0647 (10)	
H11	−0.1540	0.3766	0.5325	0.078*	
C12	−0.0007 (3)	0.3269 (2)	0.47753 (11)	0.0450 (7)	
C13	0.1247 (3)	0.2844 (2)	0.51128 (10)	0.0437 (7)	
C14	0.1214 (5)	0.2817 (3)	0.56602 (12)	0.0699 (11)	
H14	0.0327	0.3013	0.5840	0.084*	
C15	0.2500	0.2500	0.59255 (18)	0.084 (2)	
H15	0.2500	0.2500	0.6290	0.101*	
N1	0.2500	0.2500	0.48583 (10)	0.0370 (7)	
N2	0.0271 (3)	0.3210 (2)	0.42577 (9)	0.0423 (5)	
O3	0.1871 (2)	0.11853 (16)	0.35496 (7)	0.0425 (4)	
O4	0.1571 (3)	−0.0158 (2)	0.41919 (8)	0.0732 (8)	
O5	0.2500	−0.2500	0.35744 (9)	0.0370 (5)	
Zn1	0.2500	0.2500	0.404461 (14)	0.03014 (12)	

### Atomic displacement parameters (Å^2^)

**Table:**

	*U*^11^	*U*^22^	*U*^33^	*U*^12^	*U*^13^	*U*^23^
C1	0.0402 (13)	0.0278 (13)	0.0305 (12)	0.0013 (10)	−0.0055 (10)	−0.0016 (10)
C2	0.0331 (12)	0.0246 (11)	0.0259 (11)	−0.0057 (10)	0.0003 (9)	0.0003 (8)
C3	0.0432 (14)	0.0287 (13)	0.0395 (13)	−0.0004 (11)	−0.0055 (11)	0.0021 (10)
C4	0.0428 (15)	0.0493 (17)	0.0460 (16)	−0.0070 (13)	−0.0156 (13)	0.0017 (13)
C5	0.0465 (15)	0.0482 (18)	0.0505 (16)	−0.0109 (13)	−0.0102 (13)	−0.0148 (13)
C6	0.0459 (15)	0.0295 (15)	0.0528 (16)	−0.0025 (11)	−0.0031 (13)	−0.0125 (11)
C7	0.0347 (13)	0.0285 (12)	0.0301 (11)	−0.0033 (10)	0.0026 (10)	−0.0012 (9)
C8	0.0508 (18)	0.057 (2)	0.076 (2)	0.0010 (16)	−0.0083 (17)	−0.0014 (17)
C9	0.051 (2)	0.052 (2)	0.132 (4)	0.0036 (16)	−0.014 (2)	−0.005 (2)
C10	0.0508 (19)	0.047 (2)	0.145 (4)	−0.0045 (18)	0.026 (3)	−0.028 (2)
C11	0.059 (2)	0.0445 (19)	0.090 (3)	−0.0147 (16)	0.029 (2)	−0.0232 (17)
C12	0.0480 (16)	0.0293 (14)	0.0577 (17)	−0.0145 (12)	0.0178 (14)	−0.0113 (12)
C13	0.0644 (18)	0.0292 (14)	0.0377 (14)	−0.0192 (12)	0.0137 (13)	−0.0066 (10)
C14	0.120 (3)	0.051 (2)	0.0386 (17)	−0.018 (2)	0.0285 (19)	−0.0064 (13)
C15	0.160 (6)	0.065 (3)	0.028 (2)	−0.020 (4)	0.000	0.000
N1	0.0537 (18)	0.0297 (15)	0.0277 (13)	−0.0183 (15)	0.000	0.000
N2	0.0406 (12)	0.0377 (13)	0.0487 (13)	−0.0046 (10)	0.0024 (10)	−0.0027 (10)
O3	0.0645 (12)	0.0274 (9)	0.0355 (9)	−0.0114 (9)	−0.0081 (9)	−0.0014 (7)
O4	0.134 (2)	0.0536 (14)	0.0323 (11)	−0.0204 (15)	−0.0225 (12)	0.0056 (9)
O5	0.0421 (13)	0.0365 (13)	0.0324 (12)	0.0100 (12)	0.000	0.000
Zn1	0.0392 (2)	0.02603 (19)	0.02521 (19)	−0.00652 (18)	0.000	0.000

### Geometric parameters (Å, °)

**Table:**

C1—O4	1.215 (3)	C10—C11	1.353 (6)
C1—O3	1.261 (3)	C10—H10	0.9300
C1—C2	1.509 (3)	C11—C12	1.383 (4)
C2—C7	1.387 (3)	C11—H11	0.9300
C2—C3	1.394 (3)	C12—N2	1.346 (3)
C3—C4	1.377 (4)	C12—C13	1.472 (4)
C3—H3	0.9300	C13—N1	1.332 (3)
C4—C5	1.372 (4)	C13—C14	1.398 (4)
C4—H4	0.9300	C14—C15	1.362 (5)
C5—C6	1.379 (4)	C14—H14	0.9300
C5—H5	0.9300	C15—C14^i^	1.362 (5)
C6—C7	1.387 (3)	C15—H15	0.9300
C6—H6	0.9300	N1—C13^i^	1.332 (3)
C7—O5	1.399 (3)	N1—Zn1	2.078 (3)
C8—N2	1.326 (4)	N2—Zn1	2.172 (2)
C8—C9	1.393 (5)	O3—Zn1	1.9699 (17)
C8—H8	0.9300	O5—C7^ii^	1.399 (3)
C9—C10	1.360 (6)	Zn1—O3^i^	1.9699 (17)
C9—H9	0.9300	Zn1—N2^i^	2.172 (2)
			
O4—C1—O3	124.9 (2)	C12—C11—H11	120.2
O4—C1—C2	120.4 (2)	N2—C12—C11	121.3 (3)
O3—C1—C2	114.4 (2)	N2—C12—C13	115.1 (2)
C7—C2—C3	117.4 (2)	C11—C12—C13	123.5 (3)
C7—C2—C1	125.0 (2)	N1—C13—C14	119.9 (3)
C3—C2—C1	117.6 (2)	N1—C13—C12	114.8 (2)
C4—C3—C2	122.3 (2)	C14—C13—C12	125.2 (3)
C4—C3—H3	118.8	C15—C14—C13	119.0 (4)
C2—C3—H3	118.8	C15—C14—H14	120.5
C5—C4—C3	119.2 (3)	C13—C14—H14	120.5
C5—C4—H4	120.4	C14—C15—C14^i^	120.3 (4)
C3—C4—H4	120.4	C14—C15—H15	119.8
C4—C5—C6	119.9 (2)	C14^i^—C15—H15	119.8
C4—C5—H5	120.0	C13—N1—C13^i^	121.6 (3)
C6—C5—H5	120.0	C13—N1—Zn1	119.20 (16)
C5—C6—C7	120.5 (2)	C13^i^—N1—Zn1	119.20 (16)
C5—C6—H6	119.7	C8—N2—C12	118.7 (3)
C7—C6—H6	119.7	C8—N2—Zn1	126.0 (2)
C6—C7—C2	120.5 (2)	C12—N2—Zn1	115.24 (19)
C6—C7—O5	118.8 (2)	C1—O3—Zn1	118.19 (15)
C2—C7—O5	120.6 (2)	C7^ii^—O5—C7	112.7 (2)
N2—C8—C9	121.9 (4)	O3—Zn1—O3^i^	100.17 (10)
N2—C8—H8	119.1	O3—Zn1—N1	129.91 (5)
C9—C8—H8	119.1	O3^i^—Zn1—N1	129.92 (5)
C10—C9—C8	118.9 (4)	O3—Zn1—N2^i^	99.49 (9)
C10—C9—H9	120.6	O3^i^—Zn1—N2^i^	99.01 (9)
C8—C9—H9	120.6	N1—Zn1—N2^i^	75.49 (6)
C11—C10—C9	119.6 (4)	O3—Zn1—N2	99.01 (9)
C11—C10—H10	120.2	O3^i^—Zn1—N2	99.49 (9)
C9—C10—H10	120.2	N1—Zn1—N2	75.49 (6)
C10—C11—C12	119.6 (4)	N2^i^—Zn1—N2	150.99 (12)
C10—C11—H11	120.2		
			
O4—C1—C2—C7	−52.7 (4)	C9—C8—N2—C12	0.4 (5)
O3—C1—C2—C7	132.5 (3)	C9—C8—N2—Zn1	−176.8 (3)
O4—C1—C2—C3	126.2 (3)	C11—C12—N2—C8	0.4 (4)
O3—C1—C2—C3	−48.6 (3)	C13—C12—N2—C8	−177.4 (2)
C7—C2—C3—C4	0.2 (4)	C11—C12—N2—Zn1	177.9 (2)
C1—C2—C3—C4	−178.8 (2)	C13—C12—N2—Zn1	0.1 (3)
C2—C3—C4—C5	0.1 (4)	O4—C1—O3—Zn1	−1.7 (4)
C3—C4—C5—C6	0.1 (4)	C2—C1—O3—Zn1	172.90 (16)
C4—C5—C6—C7	−0.5 (4)	C6—C7—O5—C7^ii^	55.47 (19)
C5—C6—C7—C2	0.7 (4)	C2—C7—O5—C7^ii^	−123.7 (2)
C5—C6—C7—O5	−178.5 (2)	C1—O3—Zn1—O3^i^	173.4 (2)
C3—C2—C7—C6	−0.6 (3)	C1—O3—Zn1—N1	−6.6 (2)
C1—C2—C7—C6	178.3 (2)	C1—O3—Zn1—N2^i^	72.4 (2)
C3—C2—C7—O5	178.6 (2)	C1—O3—Zn1—N2	−85.2 (2)
C1—C2—C7—O5	−2.5 (4)	C13—N1—Zn1—O3	−86.96 (14)
N2—C8—C9—C10	−0.8 (6)	C13^i^—N1—Zn1—O3	93.04 (14)
C8—C9—C10—C11	0.4 (6)	C13—N1—Zn1—O3^i^	93.04 (14)
C9—C10—C11—C12	0.3 (5)	C13^i^—N1—Zn1—O3^i^	−86.96 (14)
C10—C11—C12—N2	−0.8 (4)	C13—N1—Zn1—N2^i^	−177.27 (13)
C10—C11—C12—C13	176.9 (3)	C13^i^—N1—Zn1—N2^i^	2.73 (13)
N2—C12—C13—N1	2.2 (3)	C13—N1—Zn1—N2	2.72 (13)
C11—C12—C13—N1	−175.6 (2)	C13^i^—N1—Zn1—N2	−177.27 (13)
N2—C12—C13—C14	−179.8 (3)	C8—N2—Zn1—O3	−55.0 (3)
C11—C12—C13—C14	2.5 (4)	C12—N2—Zn1—O3	127.67 (18)
N1—C13—C14—C15	3.4 (4)	C8—N2—Zn1—O3^i^	47.0 (3)
C12—C13—C14—C15	−174.5 (2)	C12—N2—Zn1—O3^i^	−130.35 (18)
C13—C14—C15—C14^i^	−1.67 (19)	C8—N2—Zn1—N1	175.9 (3)
C14—C13—N1—C13^i^	−1.7 (2)	C12—N2—Zn1—N1	−1.39 (17)
C12—C13—N1—C13^i^	176.5 (2)	C8—N2—Zn1—N2^i^	175.9 (3)
C14—C13—N1—Zn1	178.3 (2)	C12—N2—Zn1—N2^i^	−1.39 (17)
C12—C13—N1—Zn1	−3.5 (2)		

Symmetry codes: (i) −*x*+1/2, −*y*+1/2, *z*; (ii) −*x*+1/2, −*y*−1/2, *z*.
